# Whole Genome Sequencing Reveals Major Deletions in the Genome of M7, a Gamma Ray-Induced Mutant of *Trichoderma virens* That Is Repressed in Conidiation, Secondary Metabolism, and Mycoparasitism

**DOI:** 10.3389/fmicb.2020.01030

**Published:** 2020-06-12

**Authors:** Shikha Pachauri, Pramod D. Sherkhane, Vinay Kumar, Prasun K. Mukherjee

**Affiliations:** ^1^Nuclear Agriculture and Biotechnology Division, Bhabha Atomic Research Centre, Mumbai, India; ^2^Homi Bhabha National Institute, Mumbai, India; ^3^Radiation Biology & Health Sciences Division, Bhabha Atomic Research Centre, Mumbai, India

**Keywords:** *Trichoderma virens*, mutant, transcriptome, NGS, secondary metabolism

## Abstract

*Trichoderma virens* is a commercial biofungicide used in agriculture. We have earlier isolated a mutant of *T. virens* using gamma ray-induced mutagenesis. This mutant, designated as M7, is defective in morphogenesis, secondary metabolism, and mycoparasitism. The mutant does not produce conidia, and the colony is hydrophilic. M7 cannot utilize cellulose and chitin as a sole carbon source and is unable to parasitize the plant pathogens *Rhizoctonia solani* and *Pythium aphanidermatum* in confrontation assay. Several volatile (germacrenes, beta-caryophyllene, alloaromadendrene, gamma-muurolene) and non-volatile (viridin, viridiol, gliovirin, heptelidic acid) metabolites are not detected in M7. In transcriptome analysis, many genes related to secondary metabolism, carbohydrate metabolism, hydrophobicity, and transportation, among others, were found to be downregulated in the mutant. Using whole genome sequencing, we identified five deletions in the mutant genome, totaling about 250 kb (encompassing 71 predicted ORFs), which was confirmed by PCR. This study provides novel insight into genetics of morphogenesis, secondary metabolism, and mycoparasitism and eventually could lead to the identification of novel regulators of beneficial traits in plant beneficial fungi *Trichoderma* spp. We also suggest that this mutant can be developed as a microbial cell factory for the production of secondary metabolites and proteins.

## Introduction

*Trichoderma virens* is an agriculturally important fungus used for plant growth promotion and biocontrol of pathogens in commercial settings (Mukherjee et al., 2013b)^1^^,^^2^. Two strains of this fungus exist in nature: the “P” strains and the “Q” strains (Howell et al., 1993). Two isolates of *T. virens* have been widely used for genetic and biocontrol studies: Gv29-8, a “Q” strain, and IMI304061, a “P” strain (Mukhopadhyay et al., 1992; Djonović et al., 2006a, b, 2007a,b; Mukherjee et al., 2006, 2007; Bansal et al., 2018). *T. virens* is a mycoparasite on many plant pathogenic fungi and also produces several secondary metabolites, both volatile and non-volatile. Among these are gliotoxin (produced by “Q” strains), gliovirin (produced by “P” strains), viridin, viridiol, and heptelidic (koningic) acid, and several volatile sesquiterpenes, including beta-caryophyllene and germacrenes (Mukherjee et al., 2006; Crutcher et al., 2013; Zeilinger et al., 2016; Pachauri et al., 2019b). *T. virens* is also reported to induce systemic defense responses in plants (Djonović et al., 2007a; Gaderer et al., 2015; Mukherjee et al., 2018).

Whole genome of Gv29-8 and IMI304061 have been sequenced and analyzed, resulting in the identification of biosynthesis gene clusters for many unknown metabolites as well as for gliotoxin, gliovirin, viridin/viridiol, siderophores, and volatile sesquiterpenes (Kubicek et al., 2011; Crutcher et al., 2013; Sherkhane et al., 2017; Bansal et al., 2018; Mukherjee et al., 2018). Many hydrolytic enzymes and signal transduction proteins have been implicated to be involved in mycoparasitism of *Trichoderma* spp. on plant pathogenic fungi (Haran et al., 1996; Carsolio et al., 1999; Mendoza-Mendoza et al., 2003; Mukherjee et al., 2003; Druzhinina et al., 2011). A few elicitor proteins/peptides have been identified to be involved in triggering of plant defense (Djonović et al., 2006a; Viterbo et al., 2007; Mendoza-Mendoza et al., 2018). *T. virens* has also been studied for genomic presence and biological role of hydrophobins (Przylucka et al., 2006, 2017; Kubicek et al., 2008; Guzmán-Guzmán et al., 2017; Mendoza-Mendoza et al., 2018). Most of these studies involved bioinformatically identifying candidate genes, obtaining knockout mutants, and comparative phenotyping. Classical mutagenesis followed by complementation had earlier led to the discovery of several novel genes in many fungi (Mattern et al., 1992; Casselton and Zolan, 2002). In recent years, the advancements in whole genome sequencing tools have allowed for identification of mutations in classically induced mutants of fungi (McCluskey et al., 2011). The *Trichoderma reesei* strain RutC30 that is the major source of industrial cellulases was originally obtained by repeated chemical and physical mutagenesis of the wild type *T. reesei* strain QM6a. Using whole genome sequence analysis, a large deletion of 100 kb, 15 small deletions or insertions, and 223 single nucleotide variants were identified in RutC30 (Le Crom et al., 2009). We earlier obtained a mutant of *T. virens*, induced by gamma ray that does not produce conidia or detectable amounts of non-volatile secondary metabolites but has normal vegetative growth and produces excess chlamydospores (Mukherjee et al., 2006). Using SSH and this mutant, we identified a few genes involved in secondary metabolism and conidiation (Mukherjee et al., 2006; Crutcher et al., 2013; Bansal et al., 2019). Here, we describe several novel phenotypes associated with *T. virens* mutant M7, expand the repertoire of genes that are downregulated in this mutant by RNAseq and, using whole genome sequencing, attempt to identify the alterations in the genome composition of the mutant.

## Materials and Methods

### Fungal Strains and Growth Conditions

*Trichoderma virens* (IMI 304061), *Pythium aphanidermatum*, and *Rhizactonia solani* (ITCC 4110) were used from our previous studies (Mukherjee et al., 2007). The non-conidiating mutant M7 of *T. virens* was obtained from our previous study (Mukherjee et al., 2006). The fungal cultures were grown in potato dextrose medium at 28°C and maintained at −80°C for long-term storage.

### Test of Hydrophobicity

Wild-type *T. virens* and M7 mutant were grown for 5 days on PDA at 28°C, and hydrophobicity was tested by adding 0.5% aqueous aniline blue dye or water and recording the disappearance of the drop after 1 h.

### Assays for Mycoparasitism

The mycoparasitic ability of wild-type and mutant M7 was determined using confrontation assay by pairing the *Trichoderma* and the test pathogens simultaneously on the same plate, placed opposite each other. Because *T. virens* wild-type takes 4–5 days to completely overgrow these test pathogens (Mukherjee et al., 2007), ability of wild-type or mutant to overgrow and lyse the mycelia of *P. aphanidermatum* and *R. solani* was recorded after 5 days of co-inoculation. *P. aphanidermatum* and *R. solani* cell walls are primarily composed of cellulose and chitin, respectively, and hence, we assessed the ability of wild-type and M7 to utilize chitin and cellulose as a carbon source by growing them in Vogel’s minimal medium containing chitin or cellulose as the sole carbon source in liquid shake culture at 28°C and 150 rpm. For initiation of growth, 0.1% sucrose was added to the medium. All the experiments were conducted in four replicates.

### Identification of Secondary Metabolites Produced by Wild-Type and M7

The non-volatile compounds were detected in wild-type *T. virens* and M7 using thin layer chromatography as described earlier (Mukherjee and Kenerley, 2010). WT and M7 cultures were grown for 3 days in potato dextrose broth and extracted with equal volumes of ethyl acetate. The extract was dried and reconstituted in 0.1 volume methanol, and 100 μl was spotted on precoated TLC plate. TLC plate was developed in acetone:chloroform:formic acid (28:70:2) and visualized under short-wavelength UV (254 nm). The spots were eluted with 100% methanol and subjected to LC-MS/MS analysis as described earlier (Bansal et al., 2018). For confirmation, LC-MS/MS analysis of standard viridin and viridiol (a kind gift from the late Dr. C. R. Howell) and heptelidic acid (Cayman Chemical Company, United States) were also analyzed and matched with the sample.

The head space solid-phase microextraction (HS-SPME) and gas chromatography–mass spectrometry (GC–MS) technique was used for volatile compound detection in wild-type *T. virens* and M7. The instrument used for analysis was GC–MS 2010 Plus (Shimadzu, Kyoto, Japan). The GC–MS instrument was equipped with an injection port having SPME glass liner (Supelco) and RTX-5 column (5% diphenyl dimethyl polysiloxane, 10 m × 0.1 mm I.D.; Restek Corporation, Bellefonte, PA, United States). The injection port was maintained at 270°C with no solvent cut. Helium was used as a carrier gas. GC column temperature was programmed as follows: 40°C for 5 min and then increased to 200°C at 4°C min^–1^, held for 5 min and then increased to 280°C at 10°C min^–1^ with a final hold of 10 min. MS parameters were ionization voltage 70 eV, electron multiplier voltage 1 kV, and scan mode from m/z 35 to 350. Identification of the peaks was done by comparing their mass fragmentation pattern, Kovats retention indices and from the data available in the spectral (Wiley/NIST) libraries of the instrument. The experiments were conducted in three replicates and repeated once.

### Transcriptome Analysis

Wild-type and M7 were grown on PDA plates overlaid with dialysis membrane (MWCO 12 kDa) opposite of *R. solani* for 2 days. For control, wild-type *T. virens* and M7 were confronted with self. Mycelial mat from the zone of contact was harvested with a sterile spatula and frozen in liquid nitrogen and stored at −80°C until further use. Cultures were grown in four replicates, and samples were pooled before RNA extraction. RNA was extracted with TriReagent, and transcriptome sequencing was performed on Illumina HiSeq 2500 platform at M/S Scigenom, Cochin, Kerala, India, as discussed earlier (Mukherjee et al., 2019). A paired-end library was prepared and sequenced, and Trinity software was used for assembly of the cleaned reads produced by standard data preprocessing methods, including adaptor trimming, quality filtering, and end trimming. Gene expression estimation was performed by aligning the trimmed reads to the assembled transcriptome using Bowtie2 program. DESeq program was employed for differential gene expression analysis, and an in-house pipeline CANoPI (Contig Annotator Pipeline) was used for transcriptome annotation. FPKM values were considered for fold-change calculation, and log2 fold change ≥ 2 was considered for comparison of gene expression.

### Whole Genome Sequencing and Analysis

Total genomic DNA was extracted, and whole genome sequencing of the M7 mutant was performed using Illumina platform at M/S Xcelris Genomics, Ahmedabad, India. Illumina TruSeq Nano DNA HT Library Preparation Kit was used for paired end sequencing library preparation. The generated libraries were sequenced on Illumina Nextseq 500 using 2 × 150 bp chemistry. *T. virens* wild-type sequence (LQCH00000000) was used as a reference genome for M7 genome mapping using a Burrows-Wheeler Aligner (BWA) (v. 0.7.5a) program with optimized mapping parameters. The genome coverage, gene prediction, and total number of single nucleotide polymorphisms (SNPs) and insertion/deletion polymorphisms (INDELs) were analyzed and compared with the reference genome. Absence of the genes in the deleted regions was further confirmed by PCR amplification using gene specific primers (Supplementary Table S1) of representative genes using the following conditions: denaturation at 95°C for 5 min, annealing at 57°C for 45 s, extension at 72°C for 1 min, and 30 cycles.

## Results

### M7 Mutant Is Deficient in Hydrophobicity in Addition to Conidiation

**FIGURE 1:**
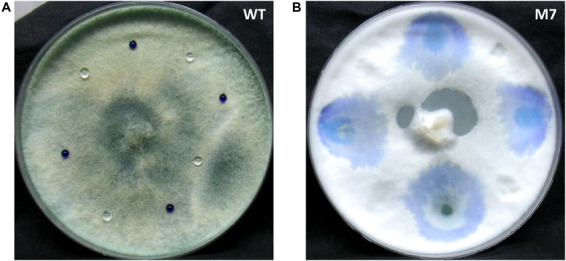
Culture of *Trichoderma virens* wild-type and M7 with aniline blue dye and water placed on it. **(A)** Wild-type. **(B)** M7.

As reported earlier (Mukherjee et al., 2006), the M7 mutant does not conidiate while wild-type *T. virens* produces green pigmented conidia (Supplementary Figure S1). The deficiency in conidiation is associated with loss of hydrophobicity. Aqueous aniline blue dye or water drops stay on the wild-type colony for several hours but diffuse through M7 colony immediately after application (Figure 1).

### M7 Is Defective in Mycoparasitism

**FIGURE 2:**
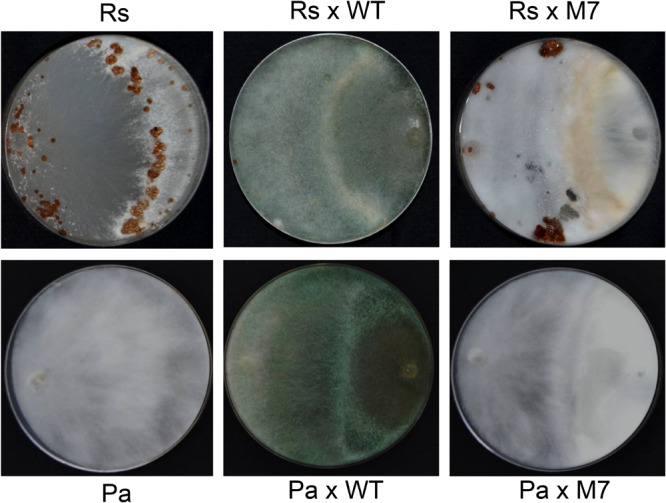
Confrontation assay demonstrating that M7 is non-mycoparasitic in nature as it cannot overgrow and lyse *Rhizoctonia solani* (Rs) and *Pythium aphanidermatum* (Pa) after 5 days of co-inoculation, and wild-type (WT) *T. virens* is able to overgrow the test pathogens.

A confrontation assay demonstrated that M7 is not able to overgrow the plant pathogens *R. solani* and *P. aphanidermatum* while wild-type *T. virens* completely overgrew the colonies in 5 days (Figure 2). Although wild-type showed mycoparasitic coiling on *R. solani* and *P. aphanidermatum*, M7 failed to do so (Supplementary Figure S2). Moreover, M7 could not utilize chitin and cellulose as a carbon source even when the medium was supplemented with 0.01% sucrose (Supplementary Figure S3).

### M7 Is Downregulated in the Biosynthesis of Secondary Metabolites

**FIGURE 3:**
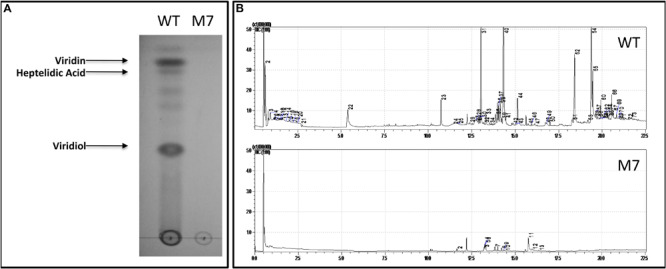
Secondary metabolite profile of *T. virens* wild-type (WT) and M7. **(A)** Thin layer chromatography analysis of WT and M7 filtrate. **(B)** GC–MS analysis of WT and M7 head-space gas. The peak numbers correspond to the volatile metabolites detected as described in Supplementary Table S2.

The TLC profile of wild-type and M7 culture filtrate shows that none of the non-volatile metabolites, including viridin, viridiol, and heptelidic acid, are detected in M7 (Figure 3A). Similarly, GC–MS analysis of head-space gas shows that M7 is downregulated in biosynthesis of volatile metabolites that are present in wild-type *T. virens*; only 13 of 73 metabolites being detected in M7 mutant (Figure 3B and Supplementary Table S2).

### Several Genes for Secondary Metabolism, Hydrolytic Enzymes, and Hydrophobicity Are Downregulated in M7

**FIGURE 4:**
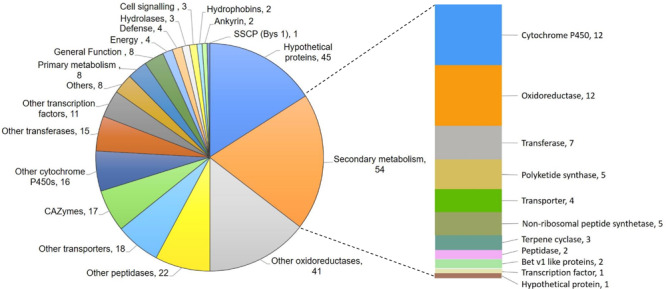
Pie chart representing genes downregulated in M7 compared to the wild-type *T. virens*.

**FIGURE 5:**
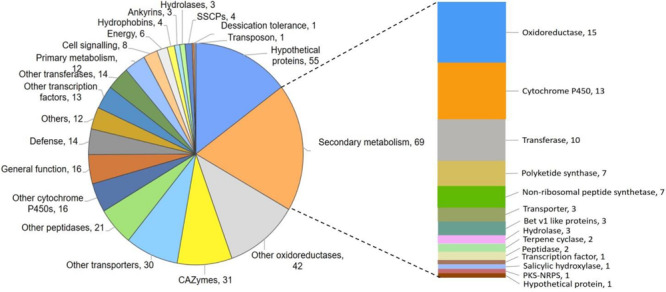
Pie chart representing genes downregulated in M7 in confrontation with *Rhizoctonia solani.*

*De novo* transcriptome analysis of wild-type and M7, while interacting with self or *R. solani* (Supplementary Figure S4), indicated that as many as 463 genes are downregulated in M7 (Supplementary Tables S3, S4, Supplementary Figures S5, S6, and Figures 4, 5). In self-confrontation, 294 genes were downregulated in M7 compared to wild-type (Supplementary Table S3). Among these are genes for secondary metabolism (including genes coding for NRPSs, PKSs, and terpene cyclases, modifying enzymes, transporters, and transcription factors), other cytochrome P450s (not associated with secondary metabolism gene clusters), carbohydrate-active enzymes (CAZymes), peptidases, and hydrophobins (Figure 4). In confrontation with the plant pathogen *R. solani*, a larger set of genes (375) were downregulated in M7, 206 genes being common under both the conditions (Supplementary Table S4 and Supplementary Figure S7). A large number of genes involved in secondary metabolism (69), carbohydrate utilization (31), and transporters (30) are downregulated in M7 in confrontation with *R. solani* (Figure 5). This is in agreement with the fact that M7 does not produce detectable amount of secondary metabolites and also does not parasitize the plant pathogens *P. aphanidermatum* and *R. solani*. Six of the genes (protein IDs. TRIVIDRAFT_49849, TRIVIDRAFT_74289, TRIVIDRAFT_74291, TRIVIDRAFT_56195, TRIVIDRAFT_23 0740, TRIVIDRAFT_53375) that are downregulated in M7 were identified in our earlier study as under-expressed in M7 in RT-PCR analysis (Mukherjee et al., 2006).

### M7 Genome Has Large Deletions

**FIGURE 6:**
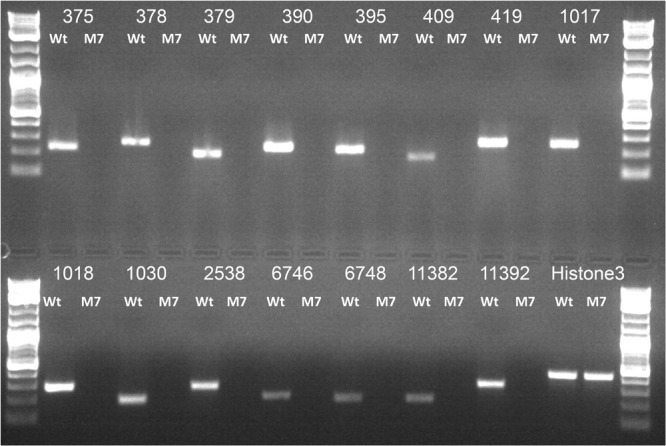
PCR analysis of representative genes that are deleted in M7, using genomic DNA as template. Histone 3 was used as a positive control. The number corresponds to gene IDs as per Table 1. Ladder: Quick-Load Purple 1 kb Plus DNA Ladder (NEB).

Whole genome sequencing of the M7 mutant was performed using the Illumina platform to generate 2.1 GB data. The paired end sequencing library was of mean size 624 bp. The generated library was sequenced and mapped to *T. virens* wild-type assembly as a reference genome. A genome alignment revealed five deletions across three scaffolds (scaffold 1, 20, and 58) totaling about 250 Kb, comprising 71 genes (Table 1). Among the genes that are absent in the M7 genome are two large secondary metabolism gene clusters (PKS6 and Tex9 clusters), eight transcription factors (two associated with secondary metabolism gene clusters), five genes for carbohydrate metabolism, two genes related to cell signaling and as many as 11 oxidoreductases (Table 1 and Supplementary Figure S8). To further confirm these deletions, PCR of representative genes was performed using wild-type and M7 genomic DNA as template. No amplification of these genes was obtained in M7, confirming the gene deletions (Figure 6). In addition, 118 SNPs and 57 small INDELs were also detected in M7 (Supplementary Table S5).

**TABLE 1:** Genes deleted in *Trichoderma virens* mutant (M7) genome.

**Gene ID (IMI 304061)**	**Size (aa)**	**Protein ID (*T. virens* Gv 29-8/ortholog)**	**Domains (NCBI CDD search)**	**Remarks/putative function**
**Deletion 1, Scaffold 1 (167 Kb)**				
CDS375	421	33554 (Tv)	Glycoside hydrolase family 28 protein	CAZyme
CDS376	558	55166 (Tv)	Aryl sulfatase	Hydrolase
CDS377	223	34822 (Tv)	Glutathione *S*-transferase family (GST)	Transferase
CDS378	430	34910 (Tv)	Transcription factor, GAL4-like Zn(II)2Cys6 (or C6 zinc) binuclear cluster DNA-binding domain	Transcription factor
CDS379	157	XP_024770685 (Th)	Transcription factor bZIP	Transcription factor
CDS380	314	34863 (Tv)	Cysteine synthase family protein	Primary metabolism
CDS381	276	137438 (Tv)	Aromatic alcohol reductase	Secondary metabolism (PKS 6 cluster)
CDS382	447	34387 (Tv)	Acyl-CoA dehydrogenase	Secondary metabolism (PKS 6 cluster)
CDS383	612	140693 (Tv)	Abhydrolase 1 (esterase/lipase)	Secondary metabolism (PKS 6 cluster)
CDS384	1241	187765 (Tv)	Multi drug resistance-associated protein (MRP), ABC transporter	Secondary metabolism (PKS 6 cluster)
CDS385	1266	34120 (Tv)	ABC transporter	Secondary metabolism (PKS 6 cluster)
CDS386	201	XP_024768020 (Th)	Adenylate forming domain, Class I superfamily and peptide synthase	Secondary metabolism (NRPS like enzyme)
CDS387	153	62551 (Tv)	No hit	Secondary metabolism (PKS 6 cluster)
CDS388	296	140688 (Tv)	Domain of unknown function (DUF3328)	Secondary metabolism (PKS 6 cluster)
CDS389	2475	62549 (Tv)	Putative polyketide synthase (PKS6)	Secondary metabolism (PKS 6 cluster)
CDS390	329	62548 (Tv)	Transcription factor, GAL4 and AflR domain-containing protein	Secondary metabolism (PKS 6 cluster)
CDS391/392	3374	70819 (Tv)	Non-ribosomal peptide synthetase (NRPS), Tex9	Secondary metabolism (Tex 9 cluster)
CDS393	378	53253 (Tv)	No hit	Secondary metabolism (Tex 9 cluster)
CDS394	590	191897 (Tv)	Multicopper oxidase with three cupredoxin domains	Secondary metabolism (Tex 9 cluster)
CDS395	505	2412 (Tv)	Transcription factor, GAL4-like Zn(II)2Cys6 (or C6 zinc) binuclear cluster DNA-binding domain and middle homology region family	Secondary metabolism (Tex 9 cluster)
CDS396	318	131309 (Tv)	Domain of unknown function (DUF3328)	Hypothetical Protein
CDS397	98	222704 (Tv)	No hit	Hypothetical Protein
CDS398	382	222705 (Tv)	Class B metal beta-lactamase	Defense
CDS399	386	OPB40689 (Tg)	No hit	Hypothetical protein
CDS400	347	53264 (Tv)	Glycoside hydrolase family 10 protein	CAZyme
CDS401	534	127080 (Tv)	Transcription factor, GAL4-like Zn(II)2Cys6 (or C6 zinc) binuclear cluster DNA-binding domain and middle homology region family	Transcription factor
CDS402	506	151748 (Tv)	Amino acid permease (GABA permease)	Transporter
CDS403	390	151736 (Tv)	Agmatinase, arginase-like and histone-like hydrolases	Primary metabolism
CDS404	394	151735 (Tv)	Glycosyltransferase family A	CAZyme
CDS405	291	191903 (Tv)	Short-chain dehydrogenases/reductases (SDR)	Oxidoreductase
CDS406	606	191904 (Tv)	Acetyltransferase (GNAT) family	Transferase
CDS407	345	191905 (Tv)	Prostaglandin dehydrogenases	Oxidoreductase
CDS408	293	216175 (Tv)	Type 1 glutamine amidotransferase (GATase1)-like domain	Transferase
CDS409	680	191907 (Tv)	Transcription factor, GAL4-like Zn(II)2Cys6 (or C6 zinc) binuclear cluster DNA-binding domain and middle homology region family	Transcription factor
CDS410	635	230771 (Tv)	Ferric reductase and NADPH oxidase (NOX) like domain-containing protein	Oxidoreductase
CDS411	78	—NA—	No hit	Hypothetical protein
CDS412	179	53545 (Tv)	Copper transporter family protein	Transporter
CDS413	431	151414 (Tv)	Transferase family	Transferase
CDS414	361	53537 (Tv)	NAD/NADP octopine/nopaline dehydrogenase and Glycerol-3-phosphate dehydrogenase like domain-containing protein	Oxidoreductase
CDS416	513	59982 (Tv)	Cytochrome P450	P450
CDS417	217	53542 (Tv)	Lipid A phosphoethanol aminetransferase	Transferase
CDS418	534	191806 (Tv)	Major facilitator superfamily	Transporter
CDS419	672	XP_013944953 (Ta)	Transcription factor middle homology region (MHR)	Transcription factor
**Deletion 2, Scaffold 1 (35 kb)**				
CDS1017	349	222457 (Tv)	Short-chain dehydrogenases/reductases (SDR)	Oxidoreductase
CDS1018	469	222632 (Tv)	Transcription factor middle homology region (MHR)	Transcription factor
CDS1019	622	53406 (Tv)	Alpha/beta hydrolases (abhydrolase)	Hydrolase
CDS1020	455	53401 (Tv)	2-Polyprenyl-6-methoxyphenol hydroxylase and related FAD-dependent oxidoreductases	Oxidoreductase
CDS1021	136	222635 (Tv)	No hit	Hypothetical protein
CDS1022	163	XP_023915254 (Qs)	Major facilitator superfamily	Transporter
CDS1023	622	201619 (Tv)	Alpha/beta hydrolases (abhydrolase)	Hydrolase
CDS1024	368	213077 (Tv)	No hit	Hypothetical protein
CDS1025	223	191847 (Tv)	Soluble inorganic pyrophosphatase	Primary metabolism
CDS1026	307	XP_024754708 (Tas)	Camphor resistance (CrcB) family protein	Defense
CDS1027	433	53420 (Tv)	Enolase superfamily	Primary metabolism
CDS1028	242	70901 (Tv)	Alginate lyase (polysaccharide lyase family 7 protein)	CAZyme
CDS1029	177	191851 (Tv)	Short-chain dehydrogenases/reductases (SDR)	Oxidoreductase
**Deletion 3, Scaffold 1 (0.3 kb)**				
CDS2538	81	65480 (Tv)	Protein tyrosine phosphatase-like protein	Post-translational modifications, signaling
**Deletion 4, Scaffold 20 (8.5 kb)**				
CDS6746	809	90966 (Tv)	Cytochrome P450 (No methyl transferase)	P450
CDS6747	667	202324 (Tv)	No hit	Hypothetical protein
CDS6748	318	51663 (Tv)	Alpha/beta hydrolase family (abhydrolase 6)	Hydrolase
**Deletion 5, Scaffold 58 (40 kb)**				
CDS11382	217	66994 (Tv)	No hit	Hypothetical protein
CDS11383	410	128312 (Tv)	Lactonase	Defense
CDS11384	261	EMR68722 (Ul)	No hit	Hypothetical protein
CDS11385	410	70730 (Tv)	No hit	Hypothetical protein
CDS11386	1167	201824 (Tv)	Catalytic domain of phospholipase D superfamily proteins	Cell signaling
CDS11387	378	152027 (Tv)	Glycosyl hydrolase families: GH43, GH62, GH32, GH68, GH117, CH130 and fungal-type cellulose-binding like domain-containing protein	CAZyme
CDS11388	390	52963 (Tv)	No hit	Hypothetical protein
CDS11389	735	KID82061 (Mg)	hAT family C-terminal dimerization region	Transposase
CDS11390	312	60120 (Tv)	S1/P1 nucleases	Primary metabolism
CDS11391	608	KAE8412673 (Ap)	Tetratricopeptide repeat	Involved in cell division
CDS11392	553	193258 (Tv)	Berberine and berberine and FAD binding like domain-containing protein	Oxidoreductase

*Tv: *Trichoderma virens*, Mg: *Metarhizium guizhouense*, Ap: *Aspergillus pseudocaelatus*, Tas: *Trichoderma asperellum*, Qs: *Quercus suber*, Ta: *Trichoderma atroviride*, Tg: *Trichoderma guizhouense*, Th: *Trichoderma harzianum*, Ul: *Eutypa lata.**

## Discussion

*Trichoderma* spp. are commercially important filamentous fungi and are used extensively in agriculture (Lorito et al., 2010; Mukherjee et al., 2013a). We had earlier isolated a non-conidiating mutant of the biocontrol agent *T. virens* that is also downregulated in secondary metabolite biosynthesis (Mukherjee et al., 2006). In the present study, we provide evidence that the mutant also lacks hydrophobicity. Hydrophobicity is imparted in fungi by small secreted cysteine-rich proteins known as hydrophobins. One hydrophobin (Trividraft_49849) was reported earlier to be downregulated in M7 (Mukherjee et al., 2006). In the transcriptome data, we noted one additional hydrophobin to be downregulated in the mutant. Hydrophobins are associated with conidiation (Wessels, 1996; Bayry et al., 2012) and loss of hydrophobicity in this non-conidiating mutant is not unexpected. *Trichoderma* hydrophobins also play important roles in attachment to root (Viterbo and Chet, 2006), elicitation of plant defense (Zhang et al., 2019), and tolerance to biotic and abiotic stresses (Przylucka et al., 2017). Hydrophobins are also involved in mycoparasitic interactions in *Trichoderma* (Guzmán-Guzmán et al., 2017). We have noticed two additional hydrophobins to be downregulated in the mutant in interaction with *R. solani* (Supplementary Table S4). These hydrophobins might, thus, be involved in mycoparasitism of *T. virens* on *R. solani*.

*Trichoderma virens* is an aggressive mycoparasite on many plant pathogenic fungi (Druzhinina et al., 2011). The mutant, however, has lost this property (Figure 2 and Supplementary Figure S2), which can be explained by the lack of upregulation of a large number of CAZymes known to be involved in parasitism on other fungi (Haran et al., 1996; Carsolio et al., 1999; Djonović et al., 2006b, 2007b). CAZymes of glycoside hydrolase 2 (GH-2), GH-3, GH-18, GH-30, GH-47, GH-55, GH-75, and GH-92 families were downregulated in M7. Furthermore, the downregulation of CAZymes of GH-16, GH-20, GH-78, and GH-79 families only during confrontation with *R. solani* suggests the role of these CAZymes in mycoparasitism. Atanasova et al. (2013a) reported upregulation of many gene families, such as those involved in metabolism, transporters, signal transduction, transcriptional regulators, defense, etc., in *T. virens* during confrontation with *R. solani*. Similarly, in our study, we have detected downregulation of different groups of genes involved in secondary metabolism (69), genes for oxidoreductases (42), CAZymes (31), peptidases (21), transporters (30), transcription factors (13), transferases (14), and defense-related genes (14) in *T. virens* during confrontation with *R. solani*. Approximately twice the number of genes for transporters (30) and carbohydrate utilization groups (31) were downregulated in M7 during confrontation with *R. solani* as compared to confrontation with self, where only 18 genes for transporter and 17 for carbohydrate utilization were downregulated (Figure 4). These results indicate that the mutant, which cannot parasitize the host fungus *R. solani*, fails to respond to its presence. The role of most of these genes in mycoparasitism is not known, and further studies on the role of these genes could throw novel insights into the phenomenon of fungus–fungus interactions.

*Trichoderma* spp. produce a large number of useful secondary metabolites with diverse biological activities (Zeilinger et al., 2016; Pachauri et al., 2019b). A large number of genes for secondary metabolism (69) were downregulated in M7 during confrontation with *R. solani*. Viridin is one of the oldest known metabolites produced by *T. virens* possessing anti-cancer and anti-fungal activities, and viridiol is a phytotoxic agent produced by irreversible enzymatic conversion of viridin (Jones and Hancock, 1987; Howell et al., 1993). Gliovirin is a ‘P’ strain-specific non-ribosomal peptide with anti-oomycete activities (Howell and Stipanovic, 1983). Non-volatile compounds, such as viridin, viridiol, and heptelidic acid, were detected by TLC followed by LC-MS/MS analysis, and gliovirin was detected by LC-MS in *T. virens* wild-type (data not presented); the mutant does not produce these metabolites. In addition, M7 is downregulated in biosynthesis of volatile organic compounds, including volatile sesquiterpenes, such as germacrenes, caryphyllene, alloaromadendrene, and gamma-muurolene, all having important biological activities (Figure 3 and Supplementary Table S2). Volatile compounds produced by *Trichoderma* spp. promote plant growth and have antimicrobial effects against plant pathogens (Vinale et al., 2008; Hung et al., 2013). In transcriptome analysis, many secondary metabolism-related genes and gene clusters were downregulated in M7 (Supplementary Tables S3, S4). The viridin biosynthesis gene cluster comprises of 21 genes (Bansal et al., 2018), out of which, 16 genes were detected to be downregulated in M7. Similarly, the gliovirin biosynthetic gene cluster has 22 genes (Sherkhane et al., 2017), of which 16 genes were detected to be downregulated in M7. All the eight genes from the “*vir*” cluster, which are associated with volatile sesquiterpene compound production (Crutcher et al., 2013; Pachauri et al., 2019a), were downregulated in M7. In addition, eight NRPSs, one NRPS/PKS, seven PKSs, and three terpene cyclase genes were downregulated in M7. The NRPS genes that are downregulated in transcriptome analysis of M7 are Tex1, Tex2, Tex5, Tex7, Tex21, Glv21, NRPS (Trividraft 50383), NRPS-like (Trividraft_222800) (NRPSs), and Tex14 (NRPS/PKS). In addition, certain genes belonging to the Tex6, Tex8, Tex10 (NRPSs), and Tex12 (NRPS/PKS) clusters were also downregulated in M7. Tex1 and Tex2 code for peptaibol synthetase (Wiest et al., 2002; Mukherjee et al., 2011). Tex10 is involved in the biosynthesis of intracellular siderophore ferricrocin while Tex21 is responsible for the biosynthesis of extracellular siderophores (Mukherjee et al., 2018). The metabolites produced by Tex5 and Tex7-NRPS genes and Tex12 and Tex14-NRPS/PKS hybrid genes are not known. Of these NRPS genes, Tex5, Tex14, and Tex21 are downregulated in M7 during confrontation with *R. solani* (Supplementary Table S4). Seven PKS genes, namely PKS4, PKS8, PKS14, and PKS17, one un-annotated PKS (Trividraft_53518), and two orthologs of *Arthroderma benhamiae* (Acc. Nos. DAA76265 and EGE05473) PKSs were downregulated in M7 during confrontation with *R. solani.* The biosynthetic products of none of these PKS genes are known. In addition, some members of PKS3 and PKS6 gene clusters were also found to be downregulated in M7. PKS4 is an ortholog of pigment-forming PKS from *T. reesei* responsible for green conidial pigment biosynthesis and is involved in mechanical stability and stress tolerance (Atanasova et al., 2013a). Because M7 does not produce conidia, it is not surprising that the pigment PKS4 gene is downregulated in M7. In addition to PKS4, another gene (*con6*) that is associated with conidiation in *Neurospora crassa* (White and Yanofsky, 1993) was also downregulated in M7.

Having noted that the mutant M7 has a deficiency in conidiation, hydrophobicity, secondary metabolism, and mycoparasitism (all traits that dictate the success of *Trichoderma* spp. as plant beneficial fungi), which was apparent from the phenotyping and transcriptome data analysis, we did a whole genome sequence analysis of the mutant vis-à-vis the wild-type strain. It was interesting to note that the M7 genome has five deletions comprising as many as 71 genes. Because the mutant was generated using gamma ray-induced mutagenesis, deletions may be expected. Two large secondary metabolism-related gene clusters (PKS6 and Tex9) are deleted in the mutant. Four genes for glycoside hydrolase (GH) are also deleted, and so are several oxidoreductases. Because the data set of genes that are under-expressed in M7 is much larger that the number of genes that are deleted in the genome, it is likely that one or a few of these genes that are deleted or affected due to other mutations (Table 1 and Supplementary Table S5) could have a pleiotropic phenotype regulating a big set of genes. Our findings identify possible candidates for future research leading to identification of master regulator gene(s) in *Trichoderma*, a biotechnologically important fungal genus. Also, because the mutant is under-regulated in secondary metabolism and a large number of carbohydrate active enzymes, it could be possible to develop this mutant as a microbial cell factory for production of secondary metabolites and proteins.

We have recently reported the isolation and characterization of a mutant (G2) of *T. virens* that is upregulated in secondary metabolite biosynthesis (Mukherjee et al., 2019). A total of 140 genes were found to be upregulated in G2 over wild-type when grown on PDA medium. Of these, as many as 45 genes are downregulated in M7, including genes for CAZymes (13), secondary metabolism (5), Cytochrome P450 (3), SSCPs (2), oxidoreductase (3), peptidases (3), metabolism (5), hydrophobins (3), hypothetical proteins (5), transporter (1), and others (2). G2 and M7, thus, seem to be two contrasting “gene-regulation” mutants of *T. virens*. Although G2 has been developed as an improved biocontrol formulation (Mukherjee et al., 2019), M7 can serve as an excellent genetic tool for understanding *Trichoderma* biology.

## Conclusion

We have identified a mutant of the plant beneficial fungus *T. virens* that is downregulated in conidiation, hydrophobicity, secondary metabolism, carbohydrate metabolism, and mycoparasitism. These traits are important for development of formulations, enzyme production, plant interactions, and biocontrol applications. By transcriptome analysis, we have also identified novel candidate genes for future research. We have also been able to identify 71 ORFs that are deleted in the mutant, which could be further studied for a role as novel regulator(s) of morphogenesis, secondary metabolism, and biocontrol properties in these plant beneficial fungal bioagents.

## Data Availability Statement

The datasets generated for this study can be found in the attached Supplementary Material. This Whole Genome Shotgun project has been deposited at DDBJ/ENA/GenBank under the accession JABENJ000000000. The version described in this manuscript is version JABENJ010000000.

## Author Contributions

SP and PS performed the laboratory experiments. PM and VK conceptualized, designed, and coordinated the studies. SP and PM analyzed the data. SP, PM, and VK wrote the manuscript.

## Conflict of Interest

The authors declare that the research was conducted in the absence of any commercial or financial relationships that could be construed as a potential conflict of interest.

## Footnotes

1 https://www.certisusa.com/pest_management_products/biofungicides/soilgard_12g_microbial_fungicide

2 http://www.acceleronsas.com/products/Pages/QuickRoots-Corn.aspx
