# Supplementary material for: Whole Genome Sequencing Reveals Major Deletions in the Genome of M7, a Gamma Ray-Induced Mutant of Trichoderma virens That Is Repressed in Conidiation, Secondary Metabolism, and Mycoparasitism
Source: Front Microbiol. 2020 Jun 12;11:1030. doi: 10.3389/fmicb.2020.01030 (PMC7303927; doi:10.3389/fmicb.2020.01030)

Supplementary Files

Frontiers in Microbiology

Whole genome sequencing reveals major deletions in the genome of M7, a gamma-ray induced mutant of *Trichoderma virens* that is repressed in conidiation, secondary metabolism and mycoparasitism

Shikha Pachauri[1,2], Pramod D. Sherkhane[1], Vinay Kumar[2,3] and Prasun K. Mukherjee[1,2]*

[1]Nuclear Agriculture and Biotechnology Division, Bhabha Atomic Research Centre, Trombay, Mumbai 400085, India

[2]Homi Bhabha National Institute, Anushaktinagar, Mumbai 400094, India

[3]Radiation Biology & Health Sciences Division, Bhabha Atomic Research Centre, Trombay, Mumbai 400085, India

*Author for correspondence (Email: prasunm@barc.gov.in; prasunmukherjee1@gmail.com)

Table S1. List of primers used present study

Primers	Sequence (5'→3')
**375F**	CACTCACTGGGCAGGATATAAT
**375R**	GCGTCATAGCCGTCTACATT
**378F**	ATCCCGGCTAAGGAACAATATC
**378R**	TGGTCGAAGCTGTGGAATG
**379F**	CCTCGATGGCAGTAACATCTC
**379R**	CACTGGAGTAAGACGTGGAATTA
**390F**	AGAATGCATCTATCTCGCAGTG
**390R**	CGACGCGACCGAATTGT
**395F**	TCCTTAACCGTCTCTTCAAACTC
**395R:**	CAAGCAAATCTAAATCCGGCAA
**409F**	ACCATCATATTTCTCAATTGAAGCC
**409R**	ATTTAGGTTATCCGCCCACAG
**419F**	TATTCAGGACATCAGCGTTCTC
**419R**	CATGGTTCCTCTTGGAAGTCA
**1017F**	TCTCACGCGAAGTCAACAA
**1017R**	ATCCTGCCGAGCAAGATAAG
**1018F**	ACTGGACTACCTGCCTTATACT
**1018R**	GCTGCTACTCAGGAATTGACTAA
**1030F**	GAACTTGAACGACGCTGCCT
**1030R**	CAGAGTCGCAGACTGCATCAA
**2538F**	AAAGGCAGCGGCTCAAA
**2538R**	GGAGGGAGTGCAGGATCT
**6746F**	ATTGCAGAGCAGGCCTTT
**6746R**	CACATCCGTCCGATTTCCTT
**6748F**	GCGGACGTGAGAAGGAAT
**6748R**	CCAGAGTACAGTCAAGGGAAC

**11382F**	CGTCAATCCGGTTGATAGTACA
**11382R**	GCCTCTATGACTCTTCTGTGATT
**11392F**	AATCCCGCATGGCGAAATA
**11392R**	CAGGGTTATCCGAATAGCAAGAA
**Histone3F**	C GTAAGCAGCTCGCCTCCAAG
**Histone3R**	CTTGCTCTGGATGGTGACAC

Tables S2-S5 are attached as Excel sheet files separately.

Figure S1. Culture of *Trichoderma virens* wild type and M7 grown on PDA for 5 days a. Front side b. Reverse side.

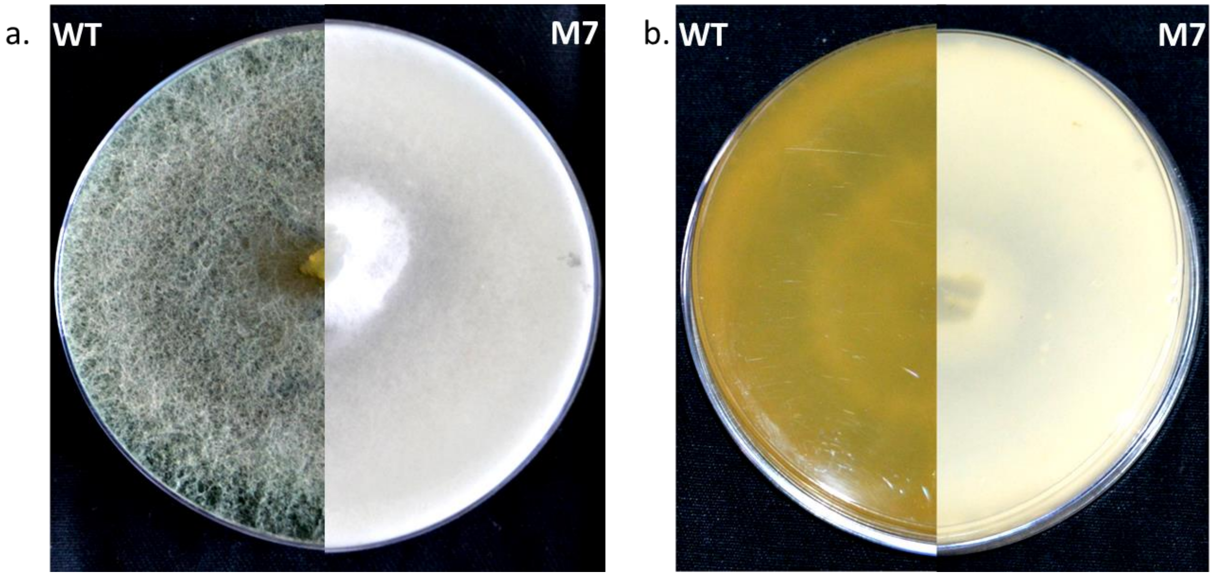

Figure S2. **Mycoparasitic coiling of *Trichoderma virens* on *Rhizoctonia solani* and *Pythium aphanidermatum*, and absence of the same in interactions with M7. Scale bar = 40 μm. Please zoom in to see the details of mycoparasitic interactions.**

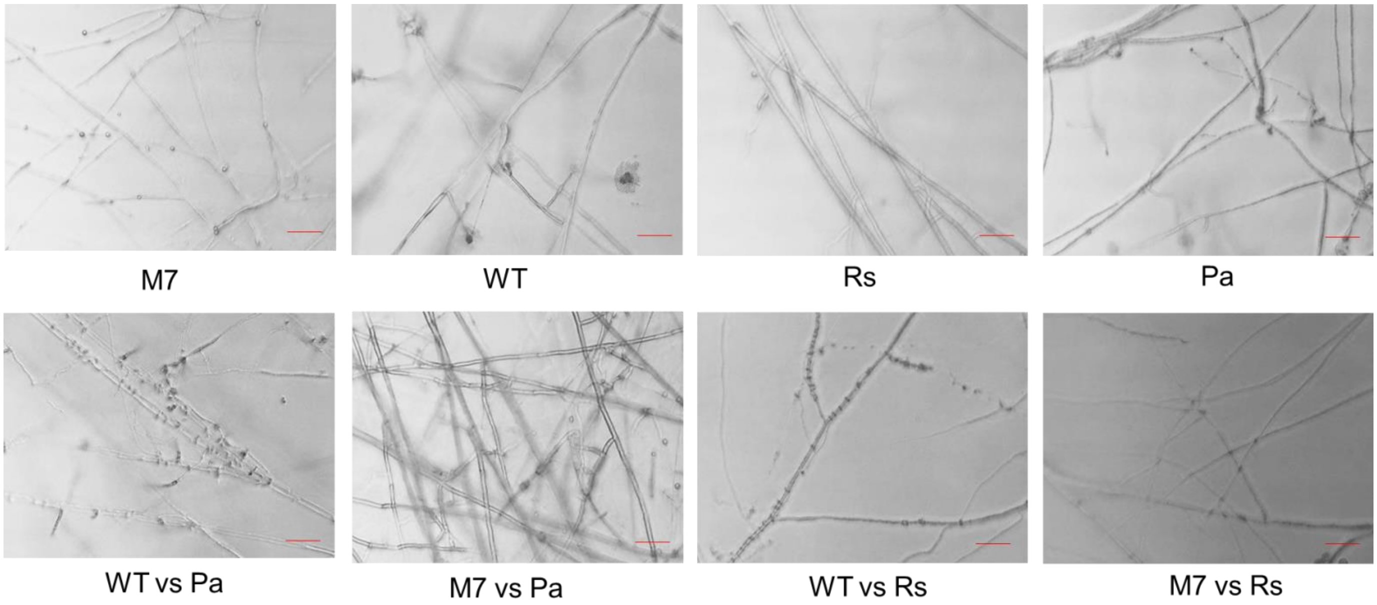

Figure S3. Seven day old culture of *T. virens* wild type (WT) and M7 in Vogel's minimal medium containing sucrose (1.5%), cellulose (plus 0.1% sucrose) or chitin (plus 0.1% sucrose). Please note that WT is able to utilize chitin and cellulose but M7 can utilize sucrose only.

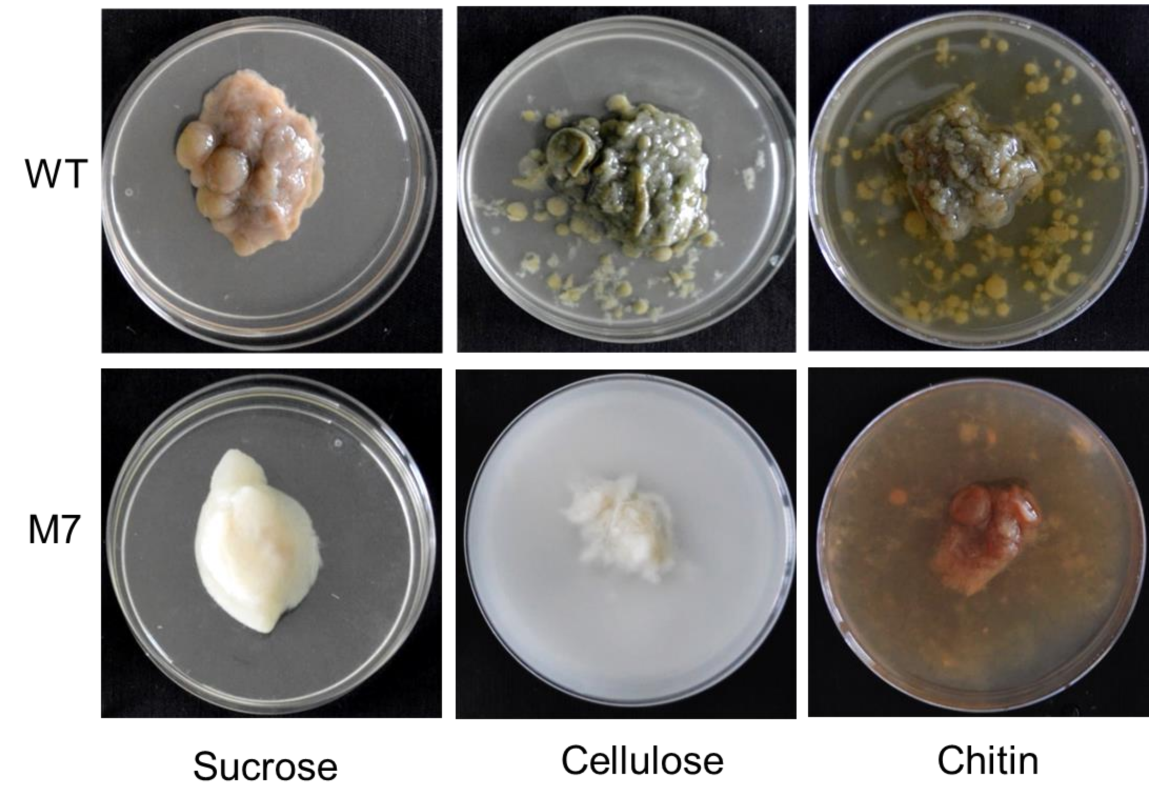

Figure S4. Confrontation of *Trichoderma virens* wild type (WT) and mutant (M7) with *Rhizoctonia solani.* Photograph was taken 48 hours after co-inoculation. The mycelial tissue from contact point were used for transcriptome analysis.

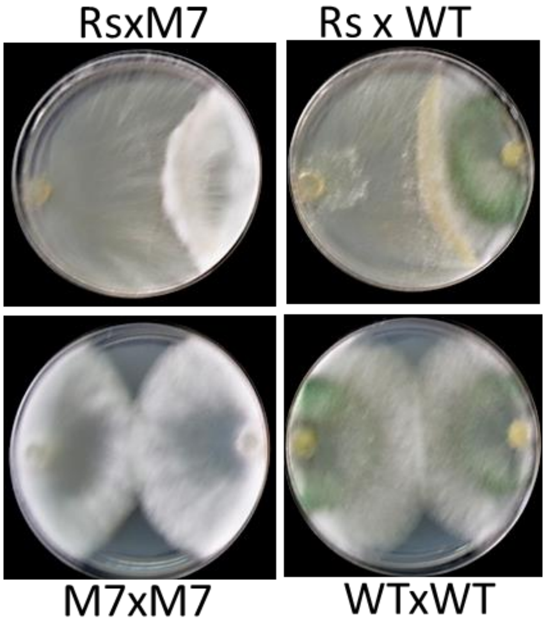

Figure. S5 Heatmap of genes downregulated in M7XM7 vs WTXWT

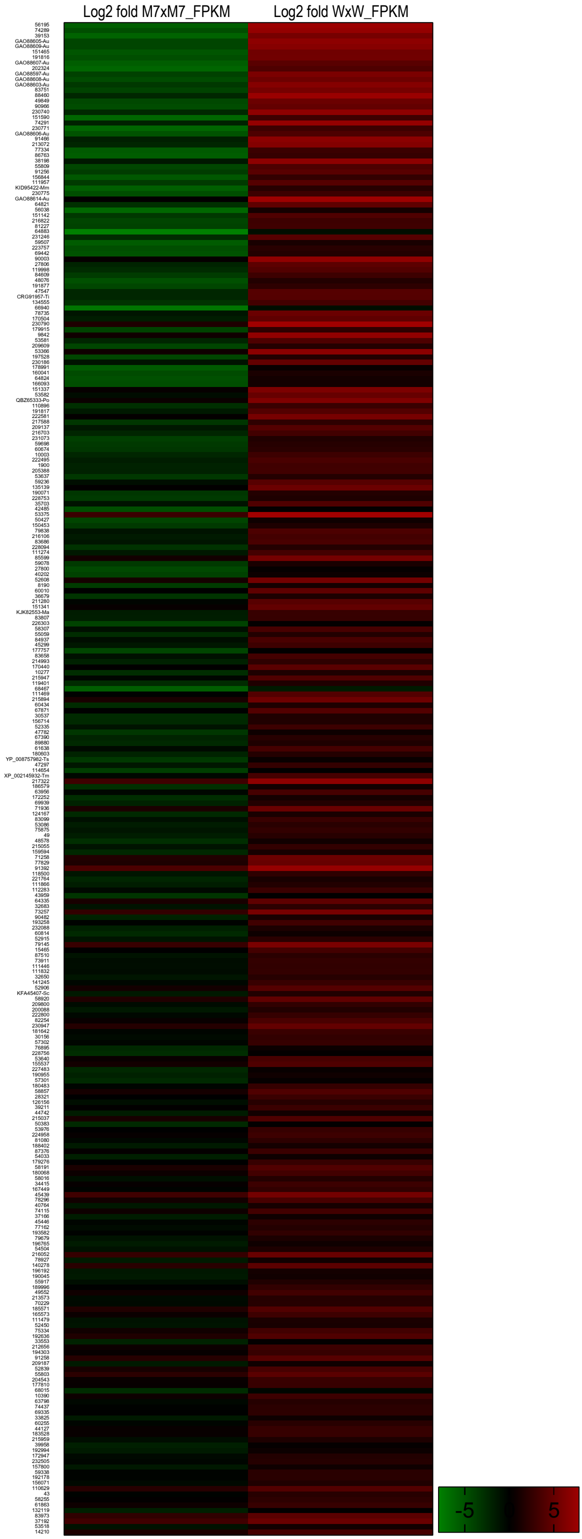

Figure. S6 Heatmap of genes downregulated in M7XRs vs WTXRs

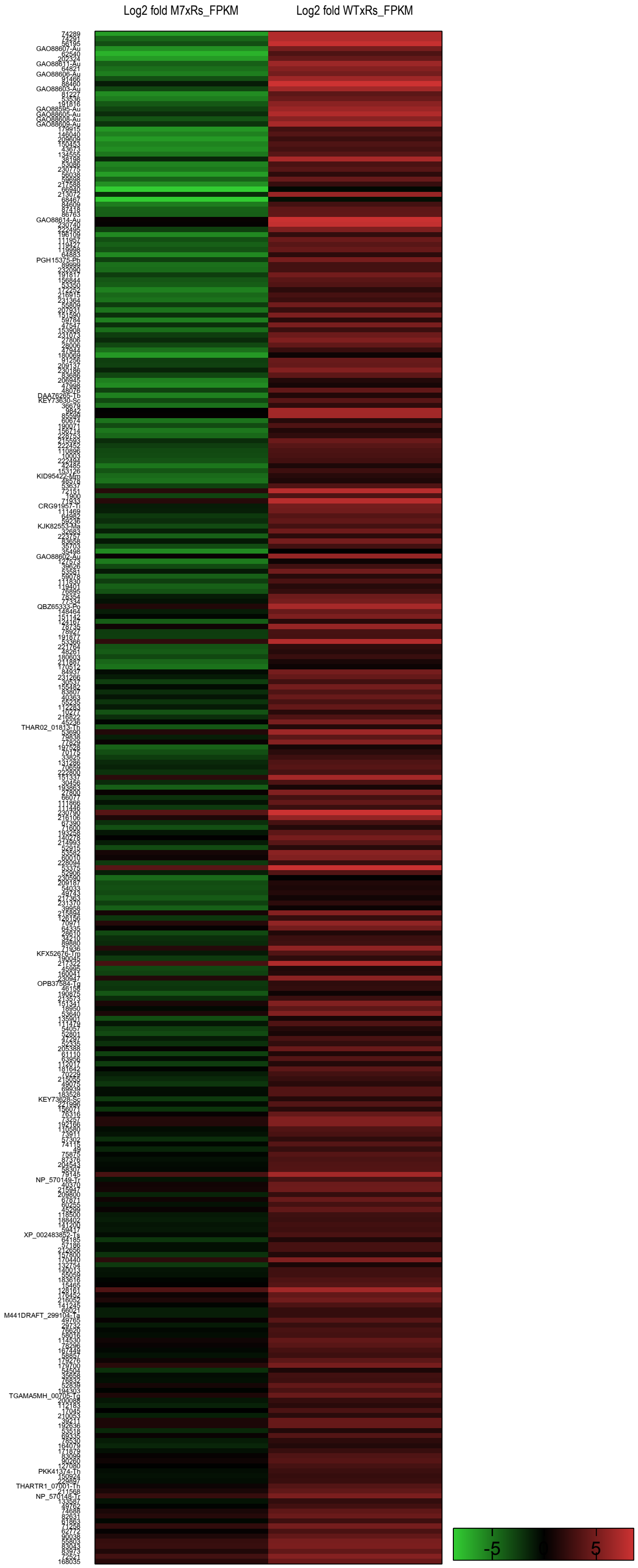

Figure S7. Venn diagramme depicting number of genes that are common and different in the two sets of RNAseq data (Wild type *Trichoderma virens* x Wild type *T. virens* vs. M7 x M7) and (Wild type *T. virens* x *Rhizoctonia solani* vs. M7 x *R. solani*).

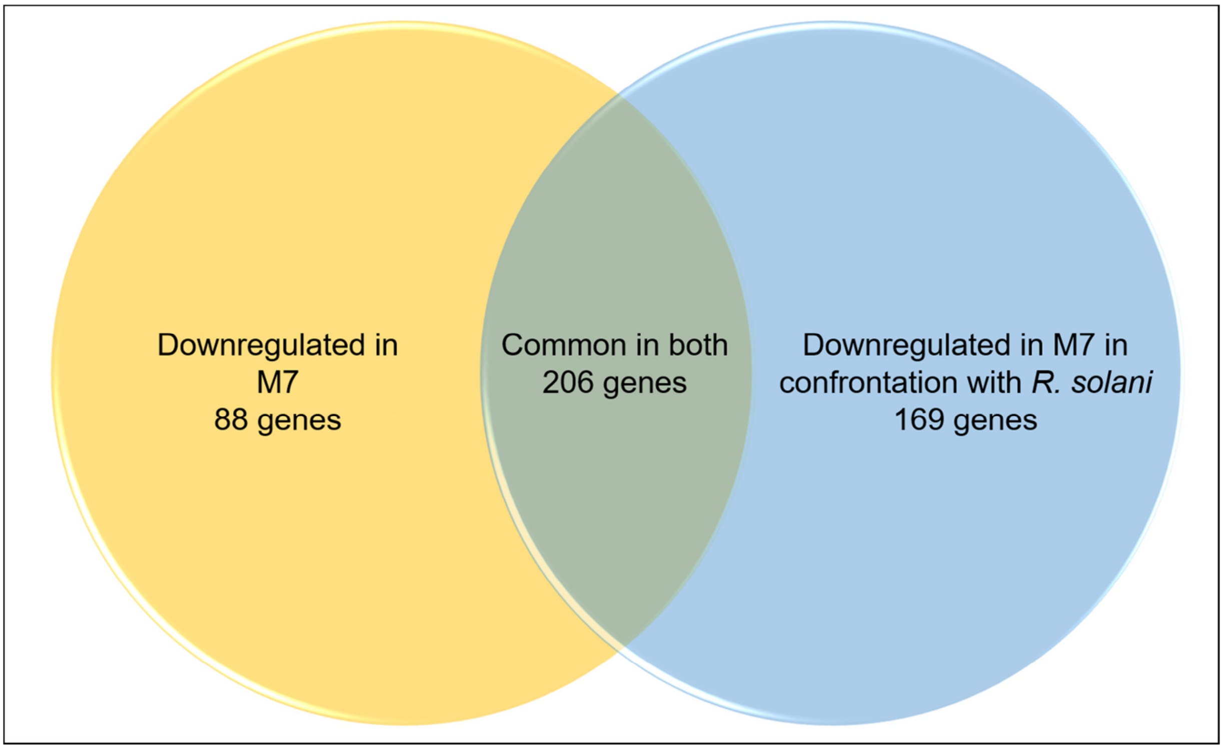

Figure S8. Pie-chart depicting putative functional classification of genes deleted in M7.

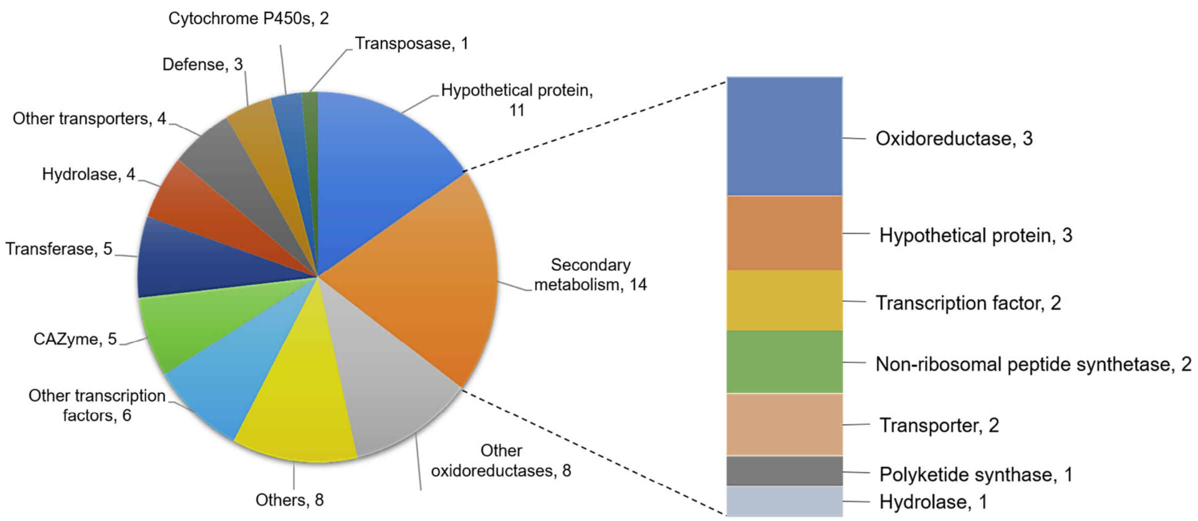

Supplement: Supplementary file 1 [file Data_Sheet_1.zip › Suppl-Mat/Data Sheet 1.pdf]
